# Knee Bone and Cartilage Segmentation Based on a 3D Deep Neural Network Using Adversarial Loss for Prior Shape Constraint

**DOI:** 10.3389/fmed.2022.792900

**Published:** 2022-05-20

**Authors:** Hao Chen, Na Zhao, Tao Tan, Yan Kang, Chuanqi Sun, Guoxi Xie, Nico Verdonschot, André Sprengers

**Affiliations:** ^1^Department of Biomechanical Engineering, University of Twente, Enschede, Netherlands; ^2^School of Instrument Science and Engineering, Southeast University, Nanjing, China; ^3^Department of Mathematics and Computer Science, Eindhoven University of Technology, Eindhoven, Netherlands; ^4^College of Health Science and Environmental Engineering, Shenzhen Technology University, Shenzhen, China; ^5^Department of Biomedical Engineering, The Sixth Affiliated Hospital, Guangzhou Medical University, Guangzhou, China; ^6^Orthopaedic Research Laboratory, Radboud University Medical Center, Nijmegen, Netherlands; ^7^Department of Biomedical Engineering and Physics, Amsterdam UMC, University of Amsterdam, Amsterdam, Netherlands

**Keywords:** cartilage segmentation, bone segmentation, MRI, deep learning, CNN

## Abstract

Fast and accurate segmentation of knee bone and cartilage on MRI images is becoming increasingly important in the orthopaedic area, as the segmentation is an essential prerequisite step to a patient-specific diagnosis, optimising implant design and preoperative and intraoperative planning. However, manual segmentation is time-intensive and subjected to inter- and intra-observer variations. Hence, in this study, a three-dimensional (3D) deep neural network using adversarial loss was proposed to automatically segment the knee bone in a resampled image volume in order to enlarge the contextual information and incorporate prior shape constraints. A restoration network was proposed to further improve the bone segmentation accuracy by restoring the bone segmentation back to the original resolution. A conventional U-Net-like network was used to segment the cartilage. The ultimate results were the combination of the bone and cartilage outcomes through post-processing. The quality of the proposed method was thoroughly assessed using various measures for the dataset from the Grand Challenge Segmentation of Knee Images 2010 (SKI10), together with a comparison with a baseline network U-Net. A fine-tuned U-Net-like network can achieve state-of-the-art results without any post-processing operations. This method achieved a total score higher than 76 in terms of the SKI10 validation dataset. This method showed to be robust to extract bone and cartilage masks from the MRI dataset, even for the pathological case.

## Introduction

Quantitative analysis of knee joint structure is a topic of increasing interest as its applications continue to broaden from direct diagnostic purposes to the implant design and preoperative and intraoperative planning. Due to the non-invasive nature and capability to discriminate cartilage from adjacent tissues, magnetic resonance imaging (MRI) is the most effective imaging device to perform knee joint analysis. However, due to the low contrast among different tissues (similar longitudinal and transverse relaxation time), image artefacts, and intensity of inhomogeneity problems in MRI (1), the accurate segmentation of the knee joint is still an open problem, especially in the knee with a degenerative disease (2).

To obtain an accurate mask for knee bone and cartilage, fully manual and semi-automatic segmentation approaches were often applied to clinical studies (3–5). Nonetheless, they were time-consuming and the reproducibility highly depends on the knowledge of experts. Hence, an automated method to segment the knee joint structure was of great interest in the past decade (6, 7). The popular methods for this aim can be divided into model-based (8–10), atlas-based (11, 12), and classification-based (1, 2, 13) methods. Although these three types of methods showed promising results to automate the knee structure segmentation, they might perform poorly in the case of high subject variability (2).

Recently, deep convolutional neural network (CNN)-based methods have achieved enormous success in biomedical imaging problems, such as classification (14) and segmentation (15–18). Regarding knee joint structure segmentation, Prasoon et al. (19) first applied the two-dimensional (2D) tri-planar CNNs (axial, coronal, and sagittal plane) to classify a pixel label (background or tibial cartilage) by providing local image patches around that pixel. Nevertheless, Ronneberger et al. (18) pointed out that there were two drawbacks to the above architecture, large redundancy and a trade-off between localisation accuracy and the use of context, and proposed a dense prediction network with skip connection, U-Net. This kind of architecture considered both the low-level and high-level features for voxel classification and was applied to the knee joint segmentation by Liu et al. (2), Zhao et al. (20), and Ambellan et al. (21). In general, the pixel-wise or voxel-wise loss, e.g., cross-entropy loss and dice loss, was utilized as the loss function for U-Net. However, there was no guarantee of the spatial consistency of the final output (22); thereafter, a further optimisation step was always required to refine the segmentation result such as deformable model (2), conditional random field (CRF) (20) and statistical shape model (SSM) (21). Although the deformable model and CRF considered the relevant spatial information to refine the segmentation, it might cause serious boundary leakage in the low-contrast regions (22). Ambellan et al. (21) proposed to utilize SSM to refine segmentation using the anatomical prior knowledge and achieved the state-of-the-art result. Nevertheless, the introduction of SSM resulted in a lot of extra calculations and the regulation was limited to the variability of the training dataset. Overall, although deep learning-based methods have been demonstrated as the state-of the-art methods in knee joint segmentation, there is still much room for improvement.

In this study, we aim to further study a three-dimensional (3D) CNN-based method to perform knee bone and cartilage segmentation. The contributions in this article are: (i) Different neural networks are proposed for bone and cartilage segmentation based on their features and a post-processing step is designed to generate the final segmentation result; (ii) the adversarial loss and a restoration network are proposed to optimize the neural network for bone segmentation and (iii) the performance of proposed method is tested on a public dataset from the Medical Image Computing and Computer-Assisted Intervention (MICCAI) Segmentation of Knee Images 2010 (SKI10) grand challenge and is fully compared with the performance of the various CNN models (3D U-Net, V-Net, nnU-Net and cascade nnU-Net) and some traditional methods.

## Materials and Methods

### Data Description

The data used in this study were from the SKI10 competition, which was focused on the knee bone and cartilage segmentation (6). The image datasets were acquired in a sagittal manner with a pixel spacing of 0.4 mm × 0.4 mm and a slice thickness of 1 mm. The total number of the knee images used in this study was 100 (60 for training and 40 for testing), and the cases of left and right knees were approximately equally distributed. Among the scans, 90% of the data were acquired at 1.5 T and the rest of the data were acquired at 3 and 1 T. The majority of data used T1 weighting and the rest of them were acquired with T2 weighting. All the images were acquired for surgery planning of partial or complete knee replacement and, therefore, a high degree of pathological deformations of the knee was included in the dataset.

### Automatic Workflow for Knee Bone and Cartilage Segmentation

**FIGURE 1:**
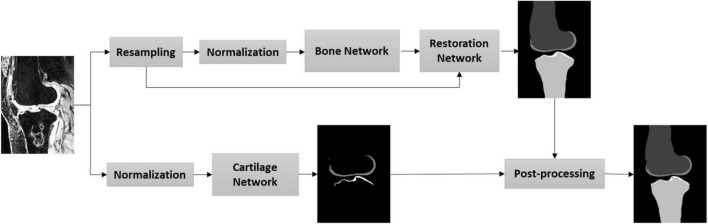
Proposed workflow for the knee bone and cartilage segmentation.

In this study, we aimed to establish a fully automatic workflow to extract knee joint structure (bone and cartilage) with highly accurate and robust segmentation, including the pathological data. Figure 1 depicts the steps of the proposed workflow. First, MRI images were resampled to enlarge the field of view by the networks; second, an image normalization method standardizes the image to a similar intensity range; third, the bone and cartilage were segmented by the bone network (Supplementary Figures 1–3 and Supplementary Table 1) in a resampled resolution; fourth, the segmented bone and cartilage masks from the bone network were restored to the original resolution through a restoration network (Supplementary Figure 4); fifth, the cartilage was segmented through a cartilage network in original resolution; last, the outputs of the cartilage network and the restoration network were post-processed for the final results.

#### Pre-processing

Our pre-processing included pixel size normalization and intensity normalization. The first pre-processing step in this study was volume resampling. One of the main challenges in medical image segmentation using deep learning is the volume size, as it is too large to feed into the networks due to the lack of the graphics processing unit (GPU) memory. A patch-wise strategy was an option to solve this issue by breaking down the volume into multiple patches (overlapping or random patches) to fit the GPU memory requirement (23). Yet, this strategy may result in a higher variance among the patches and lose the contextual information (24), especially for the large target. For the bone segmentation, we downsampled the image volume by a factor of 2 resulting in a new spacing by 0.8 × 0.8 × 2. With the resampling step, the input patch can cover more contextual information for bone segmentation. In contrast, the cartilage segmentation based on CNN is relatively sensitive to the resampling due to its small volume size. Hence, for the cartilage segmentation, we input the neural network of the image with the original size.

The second step of pre-processing was the intensity normalization. The imaging noise from the reconstruction of MRI volume, such as DC spike, results in the extreme intensity of some voxels (25). A robust intensity cut-off was selected to prevent the long intensity tail effect for both the bone and cartilage segmentation (25). In this study, the minimum and maximum cut-offs were selected as the threshold with the first and last 2% cumulative intensity histogram. Then, a following z-score strategy was adopted to normalize the intensity by subtracting the mean and dividing by the standard deviation (SD).

#### Deep Neural Network for Bone and Cartilage Segmentation

##### Architecture of the Networks

**FIGURE 2:**
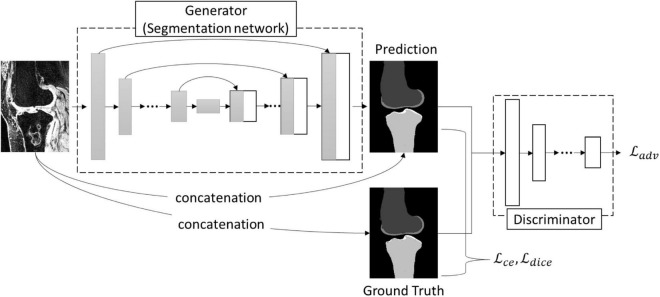
The architecture of the bone network.

Since the advent of U-Net (18), many architecture modifications have been proposed to further improve the performance of the segmentation task. However, Isensee et al. (26) demonstrated that not all of them were effective and pointed out that a typical U-Net architecture can achieve state-of-the-art results with a thorough design of adaptive pre-processing, training scheme, and inference strategy. In this study, we extended the idea of nnU-Net (26) by adding the adversarial loss to refine the segmentation and used nnU-Net as a baseline for the segmentation performance comparison. The architecture of the bone network was similar to pix2pix network (27) (Figure 2), which consisted of a generator trained for mask prediction and a discriminator trained to discriminate the produced masks (‘fake’) from ground truth labels (‘real’) (Figure 2). The framework of the generator in this study consisted of an encoding path to encode the valid features and a decoding path to perform a voxel-based classification. The encoding path contained the repeated layers of two convolutions, followed by an instance normalization, a leaky rectified linear unit, and a max pooling operation with stride 2 for downsampling. The upsampling path also contained the repeated layers of convolution, but a skip connection was adopted by a concatenation of the correspondingly cropped feature from the contraction path and the output of the up convolutions from the last layer. At the final layer, a final 1 × 1 × 1 convolution was used to map each component feature vector to the desired number of classes, and a Softmax calculation was followed at last to output a probability for each class. Both the U-Net-like (17) and V-Net-like (28) architectures were used for the generator in this study, which might result in some slight variations compared to the above description, and the detail of all the used networks in this study is summarized in the Supplementary Material.

The architecture of the discriminator of the bone network was a convolutional ‘PatchGAN’ classifier that uses the module form of convolution-batch normalization-ReLu (27). The input of the discriminator was the combination of the image patch and the corresponding segmentation patch. The detail of the architecture is provided in the Supplementary Material.

The input of the restoration network was the concatenation of the resampled image and the segmented mask from the bone network. The architecture of the restoration network consisted of two convolutional layers, followed by an upscaled deconvolutional layer, and then finally another two convolutional layers to convert the feature maps into the desired number of classes.

The architecture of the cartilage network was nnU-Net 3D at full resolution (26). The input of the cartilage network was in the original resolution, with a patch size of 160 × 192 × 64.

The details of both the cartilage network and restoration network are described in the Supplementary Material.

##### Loss Function

As Figure 2 and Equation (1) illustrate, to test the optimal loss options for a robust knee bone segmentation, the loss function, ℒ_*gen*_, used in the generator (bone network) consisted of three parts: category cross-entropy loss (ℒ_*cce*_), dice loss (ℒ_*dice*_), and adversarial loss (ℒ_*adv*_). ℒ_*cce*_ and ℒ_*dice*_ concern the low-level pixel-wise prediction, while the ℒ_*adv*_ preserves the higher-level consistency conditioned on the input.


$${ℒ}_{g\,e\,n}\,\left(x,y;\theta_{g\,e\,n},\theta_{d\,i\,s\,c}\right)=\lambda_{c\,c\,e}\,{ℒ}_{c\,c\,e}\,\left(G\,\left(x;\theta_{g\,e\,n}\right),y\right)+\lambda_{d\,i\,c\,e}\,{ℒ}_{d\,i\,c\,e}$$

(1) $$\left(G\,\left(x;\theta_{g\,e\,n}\right),y\right)+\lambda_{a\,d\,v}\,{ℒ}_{a\,d\,v}\,\left(G\,\left(x;\theta_{g\,e\,n}\right),x;\theta_{d\,i\,s\,c}\right),$$

where *x* and *y* are the input image volume and the corresponding label. λ_*cce*_, λ_*dice*_, and λ_*adv*_ are the weights for the corresponding losses and the loss is ignored if the corresponding weight sets to 0. θ_*gen*_ and θ_*disc*_ are the parameters of the networks of the generator and discriminator, respectively. The pixel-wise category cross-entropy loss is formulated as ${ℒ}_{c\,c\,e}\,\left(\hat{y},y\right)=\frac{1}{w\,h\,d}\,\sum_{i}^{w\,h\,d}\sum_{j}^{c}y_{i,j}\,\ln\left({\hat{y}}_{i,j}\right)$, where *c* represents the number of target classes and *w*, *h*, and *d* indicate the width, height, and depth of the volume patch. The pixel-wise dice loss is formulated as:${ℒ}_{d\,i\,c\,e}\,\left(\hat{y},y\right)=-\sum_{i}^{c}\frac{2\,\sum_{j}^{w\,h\,d}y_{i,j}\,\ln\left({\hat{y}}_{i,j}\right)}{\sum_{j}^{w\,h\,d}y_{i,j}^{2}+\sum_{j}^{w\,h\,d}\ln{\left({\hat{y}}_{i,j}\right)}^{2}}$. For the adversarial loss, we chose the adversarial loss of the Least Squares Generative Adversarial Network (LSGAN) (29) in this study and, therefore, is formulated as:


(2) $${ℒ}_{a\,d\,v}\,\left(x;\theta_{g\,e\,n},\theta_{d\,i\,s\,c}\right)={ℒ}_{M\,S\,E}\,\left(D\,\left(G\,\left(x;\theta_{g\,e\,n}\right);\theta_{d\,i\,s\,c}\right),1\right),$$

where ${ℒ}_{M\,S\,E}\,\left(\hat{z},z\right)={\left(\hat{z}-z\right)}^{2}$, and *x* indicates the input patch. The discriminator attempts to learn the differences between the label and prediction distributions by minimising the loss function as:


(3) $${ℒ}_{d\,i\,s\,c}\,\left(G\,\left(x;\theta_{g\,e\,n}\right),y\right)={ℒ}_{M\,S\,E}\,\left(D\,\left(G\,\left(x;\theta_{g\,e\,n}\right);\theta_{d\,i\,s\,c}\right),0\right)+{ℒ}_{M\,S\,E}\,\left(y,1\right),$$

where *x* and *y* indicate the input patch and the corresponding annotation, respectively.

For the cartilage network, the loss function is formulated as:


(4) $${ℒ}_{c\,a\,r\,t}\,\left(\hat{y},y;\theta_{c\,a\,r\,t}\right)=\lambda_{c\,c\,e}\,{ℒ}_{c\,c\,e}\,\left(\hat{y},y\right)+\lambda_{d\,i\,c\,e}\,{ℒ}_{d\,i\,c\,e}\,\left(\hat{y},y\right),$$

where *y* and $\hat{y}$ indicate ground truth and the prediction result of the cartilage network, respectively, and θ_*cart*_ indicates the parameters of the cartilage network.

For the restoration network, the loss function was formulated as:


(5) $${ℒ}_{r\,e\,s\,t\,o\,r\,e}\,\left(\hat{y},y;\theta_{r\,e\,s\,t\,o\,r\,e}\right)={ℒ}_{c\,c\,e}\,\left(\hat{y},y\right),$$

where *y* and $\hat{y}$ indicate ground truth and the prediction result of the restoration network, respectively, and θ_*restore*_ indicates the parameters of the cartilage network.

##### Training Procedure

One common challenge in deep learning training is limited training data. Data augmentation is one of the options to be taken to prevent overfitting and has been generally accepted as an add-in in the deep learning method. The data augmentation adopted in this study was random scaling (0.85–1.15), random elastic deformations, gamma correction augmentation, and random mirroring along the frontal axis (simulating the left or right knee joint).

In order to implement a fair comparison among the different architectures, the training strategy similar to a pervious study (26) was adopted. There are 6,000 training batches in an epoch. The Adam optimizer with an initial learning rate of 1 × 10^–3^ was utilized for both the generator and the discriminator in this study, and the learning rate was reduced by a factor of 5 if the loss was not improved in the last 5 epochs and the training was stopped if the loss was not improved in the last 20 epochs. The maximum epoch was limited to 500. The proposed deep CNNs were implemented in Python 3.7 using PyTorch with a 3.7-GHz Intel (R) i7 Xeon (R) E5-1620 V2 CPU and a GTX 1080 Ti graphics card with 11 GB GPU memory.

##### Inference

In the inference phase, the new input image volume was split into many sub-volume patches and input to the networks. Then, the class of each voxel was determined by the largest probability of the output probability maps from the neural network. At last, we needed to combine all the sub-volume patches back to form a full volume.

#### Post-processing

The main purpose of the post-processing is to combinate the advantages of the bone network and the cartilage network in order to generate final bone and cartilage masks. Compared to the cartilage mask from the cartilage network, the bone network could provide less mis-segmented results due to the large contextual information, however, less accurate due to lower resolution. Therefore, the output of the cartilage mask from the bone network after the restoration network was dilated by a 7 × 7 × 7 kernel, which was later used to filter the cartilage mask from the cartilage network. Finally, the ultimate output of the proposed workflow was the combination of the bone mask from the restoration network and the filtered cartilage mask of the cartilage network.

### Evaluation Design

#### Methods Designed by the Segmentation of Knee Images 2010

The evaluation method for knee bone and cartilage was different. Regarding bone segmentation, average surface distance (AvgD) and root mean square symmetric surface distance (RMSD) were proposed (6, 30).


(6) $$\text{AvgD}=\frac{1}{N_{S}+N_{R}}\,\left(\sum_{i=1}^{N_{S}}\min_{r\in \partial R}{||s_{i}-r||}_{2}+\sum_{i=1}^{N_{R}}\min_{s\in \partial S}{||r_{j}-s||}_{2}\right),$$

(7) $$\text{RMSD}=\sqrt{\frac{1}{N_{S}+N_{R}}\,\left(\sum_{i=1}^{N_{S}}\min_{r\in \partial R}{||s_{i}-r||}_{2}+\sum_{i=1}^{N_{R}}\min_{s\in \partial S}{||r_{j}-s||}_{2}\right)},$$

where ∂⁡*R* and ∂⁡*S* are the boundary of the automatic segmentation and reference segmentation, respectively, and *N_S_* and *N_R_* are the number of boundaries, respectively.

For the cartilage segmentation, volume difference (VD) and volume overlap error (VOE) were proposed (6, 21).


(8) $$\text{VD}=100\cdot \frac{\left|S\right|-\left|R\right|}{R},$$

(9) $$\text{VOE}=1-\frac{\left|S\cap R\right|}{\left|S\cup R\right|},$$

where *S* and *R* indicate automatic segmentation and reference segmentation, respectively. As indicated by Heimann et al. (6), the cartilage boundaries to the sides were not always accurate; regions of interest (ROIs) for cartilage mask comparison were used in the above calculation.

#### Dice Similarity Coefficient

The Dice similarity coefficient (DSC) score is defined as:


(10) $$\text{DSC}=\frac{2\,T_{P}}{2\,T_{P}+F_{P}+F_{N}},$$

(11) $$\text{Sensitivity}=\frac{T_{P}}{T_{P}+F_{N}},$$

(12) $$\text{Specificity}=\frac{T_{N}}{T_{N}+F_{P}},$$

where *T_P_* is true positive,*T_N_* is false negative, *F_P_* is false positive, and *F_N_* is false negative. The thickness difference is calculated by the thickness difference from each vertex along the normal vector between automated and manual segmentation masks.

## Results

Table 1 summarizes the results of previous studies (2, 9, 10, 12, 21, 30), baseline networks [nnU-Net (26, 31), including the 2D version, 3D full-resolution version, and 3D low-resolution version], and the proposed methods for the SKI10 validation dataset in terms of the SKI10 metrics (6). The bone and cartilage segmentation results with proposed networks reached a total score of 76.2 ± 7.6, which was for the first time higher than 75 using the validation dataset [the second rater’s score was 75 in a previous study (6)]. Overall, the results of deep learning-based methods outperformed the traditional methods [atlas based (12) and statistical shape-based methods (9, 10, 30)]. The new baseline (nnU-Net) could achieve state-of-the-art results without any post-processing. Still, the proposed method outperformed the baseline.

**TABLE 1:** Comparison of automatic segmentation methods based on the Segmentation of Knee Images 2010 (SKI10) validation data.

		Femur bone		Tibia bone		Femur cartilage		Tibia cartilage	
Team (reference)	Total score	AvgD (mm)	RMSD (mm)	AvgD (mm)	RMSD (mm)	VOE (%)	VD (%)	VOE (%)	VD (%)
Vincent et al. (10)	52.3 ± 8.6	0.88 ± 0.24	1.49 ± 0.44	0.74 ± 0.21	1.21 ± 0.34	36.3 ± 5.3	−25.2 ± 10.1	34.6 ± 7.9	74.0 ± 7.7
Seim et al. (9)	54.4 ± 8.8	1.02 ± 0.22	1.54 ± 0.30	0.84 ± 0.19	1.24 ± 0.28	34.0 ± 12.7	7.7 ± 19.2	29.2 ± 8.6	−2.7 ± 18.2
Shan et al. (12)	40.0 ± 7.7	–	–	–	–	–	–	–	–
*Liu et al. (2)	64.1 ± 9.5	0.56 ± 0.12	1.08 ± 0.21	0.50 ± 0.14	1.09 ± 0.28	28.4 ± 6.9	8.1 ± 12.3	33.1 ± 7.1	−1.2 ± 17.4
Dam et al. (30)	67.1 ± 8.0	0.68 ± 0.22	1.25 ± 0.41	0.50 ± 0.18	0.91 ± 0.35	26.9 ± 6.0	0.8 ± 13.5	25.1 ± 6.7	0.41 ± 13.4
*Ambellan et al. (21)	74.0 ± 7.7	0.43 ± 0.13	0.74 ± 0.27	0.35 ± 0.07	0.59 ± 0.19	20.99 ± 5.08	7.18 ± 10.51	19.06 ± 5.18	4.29 ± 12.34
3D U-net (17)	48.1 ± 12.3	1.77 ± 1.85	5.24 ± 3.99	2.60 ± 2.59	7.50 ± 5.29	23.80 ± 7.25	−5.45 ± 8.37	20.60 ± 6.40	5.48 ± 15.11
V-Net (28)	55.7 ± 10.7	0.88 ± 0.61	3.36 ± 2.46	1.04 ± 0.95	4.23 ± 3.53	21.91 ± 4.48	1.17 ± 9.14	20.08 ± 5.62	6.12 ± 16.57
Cascade nnU-Net (26)	75.4 ± 8.1	0.37 ± 0.12	0.63 ± 0.29	0.32 ± 0.15	0.57 ± 0.39	22.71 ± 4.88	1.76 ± 10.03	21.21 ± 5.83	7.05 ± 13.66
*nnU-Net 2D (26)	73.4 ± 10.7	0.37 ± 0.15	0.69 ± 0.35	0.38 ± 0.27	0.80 ± 0.77	21.34 ± 5.59	4.49 ± 11.46	21.43 ± 5.67	5.74 ± 13.41
*nnU-Net 3D full res (26)	72.5 ± 14.2	0.56 ± 1.00	1.67 ± 2.96	0.44 ± 0.57	1.34 ± 2.46	19.45 ± 5.06	6.79 ± 10.29	18.09 ± 5.09	8.32 ± 11.31
*nnU-Net 3D low res (26)	75.3 ± 9.3	0.35 ± 0.12	0.65 ± 0.30	0.34 ± 0.23	0.75 ± 1.19	21.72 ± 4.70	3.66 ± 12.14	21.78 ± 5.39	6.58 ± 12.11
*Proposed method	76.2 ± 7.6	0.38 ± 0.15	0.69 ± 0.37	0.29 ± 0.07	0.52 ± 0.12	19.45 ± 5.06	6.78 ± 10.29	18.09 ± 5.09	8.32 ± 11.31

* indicates the deep learning-related method; ‘res’ indicates resolution.

Moreover, Table 2 shows the accuracy evaluation for the SKI10 dataset between the baseline networks and the proposed methods in terms of the DSC, sensitivity, and specificity. For the cartilage result, the DSC is only calculated in the defined ROI according to a previous study (6). The DSC scores of the proposed method are 0.98 ± 0.01, 0.98 ± 0.01, 0.89 ± 0.03, and 0.88 ± 0.03 for femur bone, tibia bone, femur cartilage, and tibia cartilage, respectively. Overall, the performance of the proposed methods achieved the highest score.

**TABLE 2:** Segmentation accuracy for the SKI10 validation dataset between baseline networks and the proposed methods.

	Femur bone			Tibia bone			Femur cartilage			Tibia cartilage		
	DSC	Sens	Spec	DSC	Sens	Spec	DSC	Sens	Spec	DSC	Sens	Spec
2D	0.98 ± 0.01	0.98 ± 0.02	1.00 ± 0.00	0.98 ± 0.02	0.98 ± 0.03	1.00 ± 0.00	0.88 ± 0.04	0.90 ± 0.04	1.00 ± 0.00	0.86 ± 0.04	0.89 ± 0.06	1.00 ± 0.00
3D F	0.98 ± 0.01	0.98 ± 0.01	1.00 ± 0.00	0.98 ± 0.02	0.98 ± 0.03	1.00 ± 0.00	0.89 ± 0.03	0.92 ± 0.04	1.00 ± 0.00	0.88 ± 0.03	0.92 ± 0.04	1.00 ± 0.00
3D L	0.98 ± 0.01	0.98 ± 0.01	1.00 ± 0.00	0.98 ± 0.01	0.98 ± 0.02	1.00 ± 0.00	0.88 ± 0.03	0.89 ± 0.05	1.00 ± 0.00	0.86 ± 0.04	0.89 ± 0.05	1.00 ± 0.00
Proposed	0.98 ± 0.01	0.98 ± 0.01	1.00 ± 0.00	0.98 ± 0.01	0.98 ± 0.01	1.00 ± 0.00	0.89 ± 0.03	0.92 ± 0.04	1.00 ± 0.00	0.88 ± 0.03	0.92 ± 0.04	1.00 ± 0.00

*Two-dimensional (2D), nnU-Net 2D; three-dimensional (3D) F, nnU-Net 3D full resolution; 3D L, nnU-Net 3D low resolution; DSC, dice similarity coefficient; Sens, sensitivity; Spec, specificity.*

**FIGURE 3:**
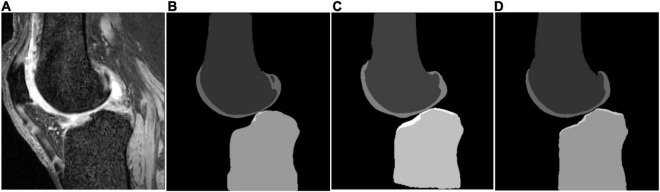
Segmentation results based on different schemes: **(A)** sagittal slice of the image; **(B)** ground truth; **(C)** nnU-Net two-dimensional (2D); and **(D)** proposed method.

**FIGURE 4:**
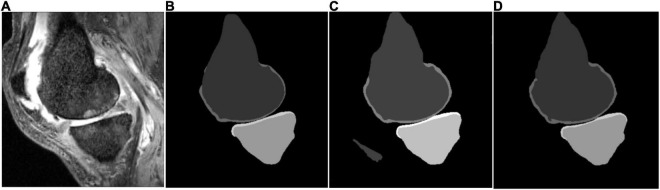
Segmentation results based on different schemes: **(A)** sagittal slice of the image; **(B)** ground truth; **(C)** nnU-Net three-dimensional (3D) full resolution; and **(D)** proposed method.

**FIGURE 5:**
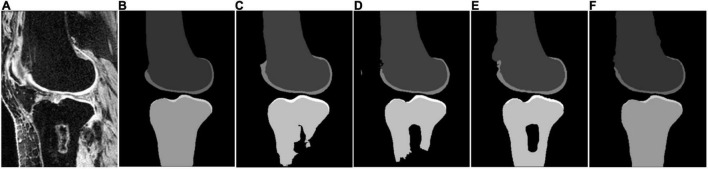
Segmentation results based on different schemes: **(A)** sagittal slice of the image; **(B)** ground truth; **(C)** nnU-Net 2D; **(D)** nnU-Net 3D full; **(E)** nnU-Net 3D low; and **(F)** proposed method.

Some segmentation results on the SKI10 validation set are shown in Figures 3–5, which compared the baseline networks with the proposed method. The results of nnU-Net 2D might mis-segment the low-contrast region (bottom of Figure 3C), while the result of nnU-Net 3D full resolution might mis-segment some of the unrelated regions (left bottom of Figure 4C). A segmentation result of knee joint image with specific pathological tissue is given in Figure 5. All the baseline networks failed to segment it successfully and the proposed method with the adversarial loss showed a robust result (Figure 5F).

In addition, an ablation study about the loss function selection is shown in Table 3. The proposed loss function is capable of improving the segmentation performance.

**TABLE 3:** Results of the different loss functions based on the proposed network.

		Femur bone		Tibia bone		Femur cartilage		Tibia cartilage	
Loss	Total score	AvgD (mm)	RMSD (mm)	AvgD (mm)	RMSD (mm)	VOE (%)	VD (%)	VOE (%)	VD (%)
CE loss	73.85 ± 9.37	0.43 ± 0.36	1.17 ± 1.88	0.48 ± 0.79	1.25 ± 2.64	21.46 ± 5.17	4.44 ± 9.83	18.44 ± 5.07	6.11 ± 13.49
SD loss	67.54 ± 14.78	0.92 ± 1.50	2.57 ± 4.15	0.82 ± 1.85	2.08 ± 4.57	19.89 ± 5.70	7.65 ± 10.01	18.64 ± 6.49	13.08 ± 13.04
CE loss + SD loss	74.38 ± 10.39	0.38 ± 0.23	1.08 ± 1.33	0.31 ± 0.30	0.58 ± 0.71	20.00 ± 5.63	6.60 ± 9.98	18.62 ± 5.95	10.75 ± 13.41
Proposed loss	76.2 ± 7.6	0.38 ± 0.15	0.69 ± 0.37	0.29 ± 0.07	0.52 ± 0.12	19.45 ± 5.06	6.78 ± 10.29	18.09 ± 5.09	8.32 ± 11.31

Computation time for the whole segmentation pipeline for one subject is measured as around 1 min on a consumer-grade workstation (CPU: Intel Xeon E5 2.3 GHz; GPU: GeForce GTX 1080 Ti).

## Discussion

In this study, we presented an end-to-end deep learning-based workflow for knee bone and cartilage segmentation and evaluated the workflow thoroughly on a published dataset, the SKI10 (6). It was the first time that a total score greater than 76 was achieved on the SKI10 validation dataset, which was comparable to the inter-observer variability of two expert readers (6).

The attempt of applying deep learning-based methods to the knee bone and cartilage segmentation was not new and has achieved a lot of state-of-the-art results (2, 21). Nevertheless, most of the previous attempts added a post-processing step [deformable model (2), conditional random field (CRF) (20) and statistical shape model (SSM) (21)] to refine the outcome of the deep learning methods on the area of false segmentation. The main reason behind this is that the information of highly patient-specific areas might not be derived from the training dataset (21). To confirm the necessity of the post-processing, a generic U-Net architecture with fine-tuned hyper-parameter (31) was tested in this study as the baseline. State-of-the-art results can be achieved using the simple nnU-Net architectures (see Table 1). Nonetheless, due to the loss of *Z*-axis information and contextual information, the performance of bone segmentation of generic 2D nnU-Net and 3D nnU-Net full resolution might perform poorly in the low-contrast region (Figure 3C), bone- or cartilage-like region (Figure 4C), and region with pathological case (Figures 5C,D). Table 1 has shown a good bone segmentation result using the 3D nnU-Net low-resolution version but not in cartilage segmentation. This is because the target volume of cartilage is relatively small, which resulted in the loss of the cartilage information, especially in the pathological area. In this sense, whether resampling the image volume is necessary to improve the segmentation performance should be considered carefully based on the size of the target and the memory of the GPU.

Moreover, Figures 5C–E have shown that all the nnU-Net architectures fail to segment the bone with the specific pathological feature, which demonstrates the necessity of post-processing from previous studies. In this study, we introduced the adversarial loss to serve as a shape regulation penalty to improve bone segmentation. Although the adversarial loss (32) has been proposed to improve the segmentation performance previously, to the best of our knowledge, it was the first time to serve as a shape consistency term to apply to knee bone MRI image segmentation. Figure 5F has shown that the introduction of adversarial loss results in state-of-the-art results for bone segmentation despite the pathological case. In addition, a possible alternative method to improve the segmentation performance for the pathological case is to increase the training set size, especially for the pathological case.

This study has a number of limitations. First of all, due to the limited memory of Nvidia 1080 Ti, the number of feature channels of the first layer in nnU-Net experiments is 20 rather than 30 as stated in previous research (31). Further experiments with a better GPU should be implemented to investigate the performance influence of the number of feature channels. An additional limitation is that there are still a lot of ablation studies, which can be implemented to discuss the segmentation performance based on different choices of hyper-parameters. Nevertheless, we believe that the experiment results are enough to share with the community to help the development of fully automatic segmentation of the knee joint. Moreover, the bone segmentation was segmented in a relatively lower resolution in order to enlarge context information. Isensee et al. (26) proposed a cascaded mode to further improve the low-resolution segmentations. However, the training data for these two networks should be different; otherwise, it will easily result in an over-fitted network. As Isensee (33) stated that the cascaded mode was not so much better than the 3d_lowres and 3d_full_res mode in most cases, we believe that the results of 3d_lowres and 3d_full_res are sufficient to be a baseline and we will add the comparison with a cascaded mode in the future when a more annotated dataset is available.

## Conclusion

To conclude, we presented a robust pipeline to segment the knee bone and cartilage. The result of the proposed method is the first time achieved more than 76 in a well-known dataset, the SKI10 validation, to the best of our knowledge. The lower-resolution strategy and the introduction of adversarial loss improve the shape consistency of the bone segmentation, while a fine-tuned V-Net network was further boosted to achieve a promising result for the cartilage segmentation. Future studies will include segmentation for more knee joint structures such as ligaments and menisci.

## Data Availability Statement

The original contributions presented in the study are included in the article/Supplementary Material, further inquiries can be directed to the corresponding authors.

## Author Contributions

HC, NZ, TT, and AS conceived the study. HC, NZ, and TT designed the experiments. HC implemented the model and experiments. HC and AS wrote the manuscript. YK, NV, and AS helped to supervise the study. CS and GX helped to test the segmentation performance based on the previous CNN methods (3D U-Net, V-Net, etc.). All authors discussed the results and contributed to the final version of the manuscript.

## Conflict of Interest

The authors declare that the research was conducted in the absence of any commercial or financial relationships that could be construed as a potential conflict of interest.

## Publisher’s Note

All claims expressed in this article are solely those of the authors and do not necessarily represent those of their affiliated organizations, or those of the publisher, the editors and the reviewers. Any product that may be evaluated in this article, or claim that may be made by its manufacturer, is not guaranteed or endorsed by the publisher.
