# Supplementary material for: Knee Bone and Cartilage Segmentation Based on a 3D Deep Neural Network Using Adversarial Loss for Prior Shape Constraint
Source: Front Med (Lausanne). 2022 May 20;9:792900. doi: 10.3389/fmed.2022.792900 (PMC9163741; doi:10.3389/fmed.2022.792900)
Supplement: Supplementary file 1 [file Data_Sheet_1.docx]

Supplementary Material

# Supplementary Figures


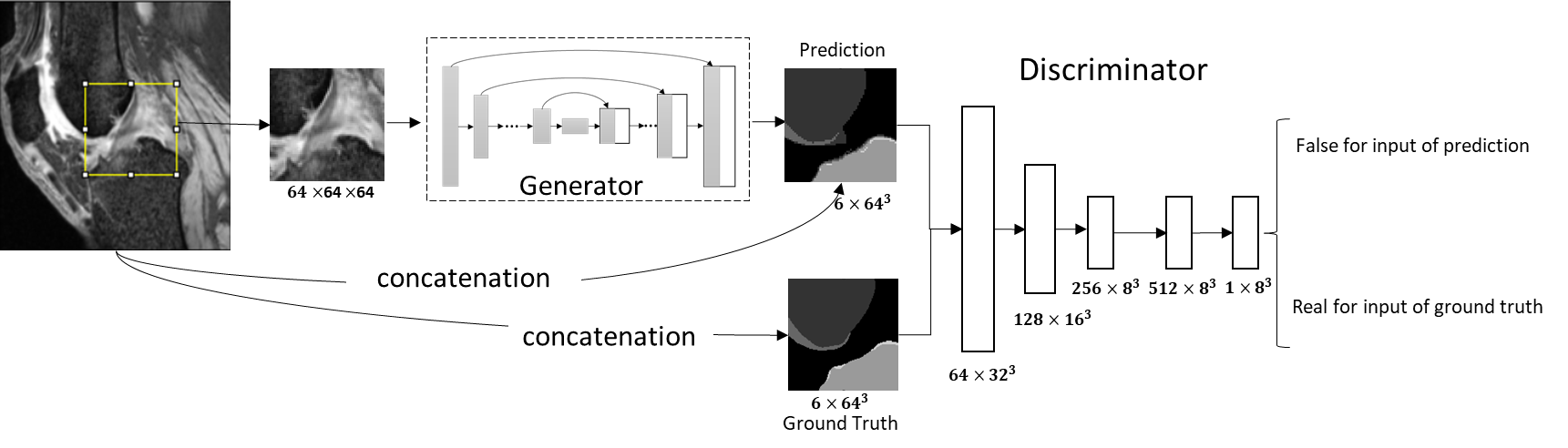


**Supplementary Figure 1.** The architecture of the bone network (the architecture of the generator is depicted in Figure Supplementary Figure 2; In the discriminator, first two layers of discriminator follow the module form of Conv3-LeakyRelu, the third and fourth layers follow module form of Conv3-InstanceNorm-LeakyRelu, and the last layer is with only Conv3).


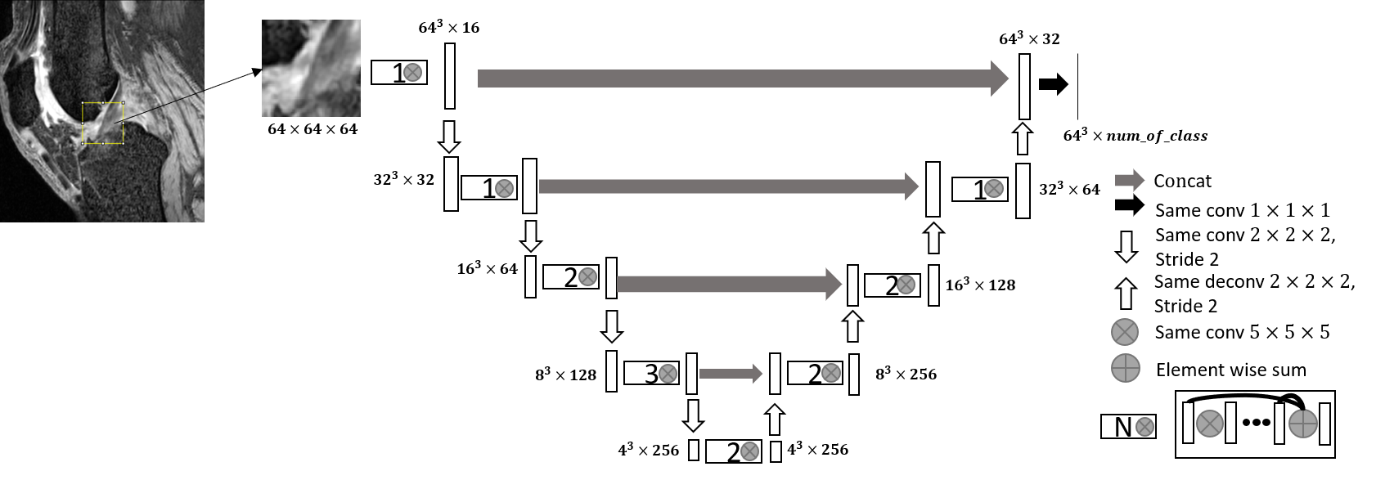


**Supplementary Figure 2.** The detail architecture of the generator of bone network.


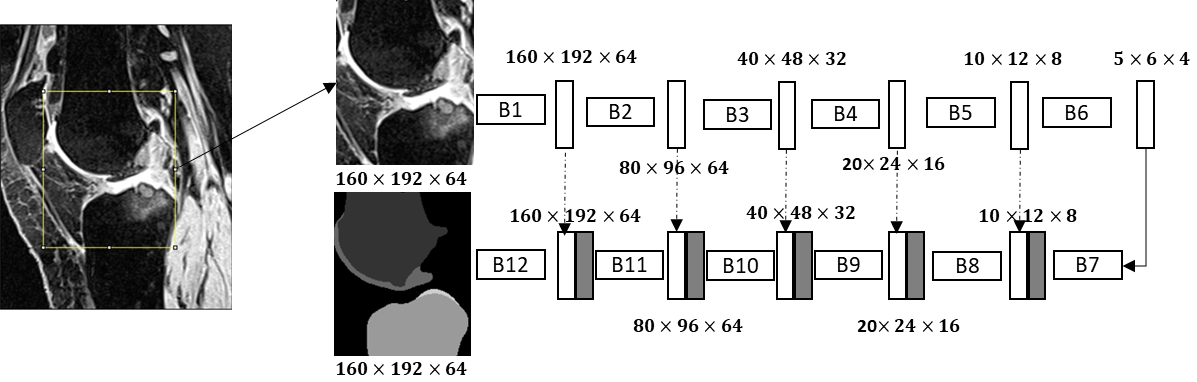


**Supplementary Figure 3.** The architecture of the cartilage network (The layers of the block follow the module form of Conv3-InstanceNorm-LeakyRelu).


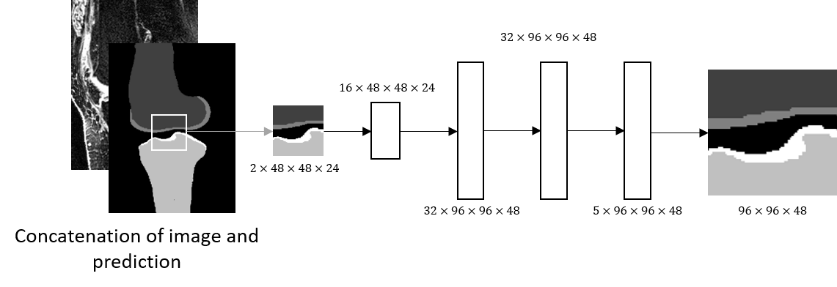


**Supplementary Figure 4.** The architecture of the restoration network (the first, third and fourth layers follow the module form of Conv3-BatchNorm-RELU; the second layer follows the module form of Deconv3-BatchNorm-RELU).

# Supplementary Table

**Supplementary Table 1.** Parametric setting of blocks in neural network in Supplementary Figure 3.

| Block | Kernel | Stride | Padding | Feature | Block | Kernel | Stride | Padding | Feature |
| --- | --- | --- | --- | --- | --- | --- | --- | --- | --- |
| B1 | Conv (3,3,1) | (1,1,1) | (1,1,0) | 1-20 | B8 | Conv (3,3,3) | (1,1,1) | (1,1,1) | 640-320 |
|  | Conv (3,3,1) | (1,1,1) | (1,1,0) | 20-20 |  | Conv (3,3,3) | (1,1,1) | (1,1,1) | 320-320 |
| B2 | Conv (3,3,3) | (2,2,1) | (1,1,1) | 20-40 |  | Deconv (2,2,2) | (2,2,2) | - | 320-160 |
|  | Conv (3,3,3) | (1,1,1) | (1,1,1) | 40-40 | B9 | Conv (3,3,3) | (1,1,1) | (1,1,1) | 320-160 |
| B3 | Conv (3,3,3) | (2,2,2) | (1,1,1) | 40-80 |  | Conv (3,3,3) | (1,1,1) | (1,1,1) | 160-160 |
|  | Conv (3,3,3) | (1,1,1) | (1,1,1) | 80-80 |  | Deconv (2,2,2) | (2,2,2) | - | 160-80 |
| B4 | Conv (3,3,3) | (2,2,2) | (1,1,1) | 80-160 | B10 | Conv (3,3,3) | (1,1,1) | (1,1,1) | 160-80 |
|  | Conv (3,3,3) | (1,1,1) | (1,1,1) | 160-160 |  | Conv (3,3,3) | (1,1,1) | (1,1,1) | 80-80 |
| B5 | Conv (3,3,3) | (2,2,2) | (1,1,1) | 160-320 |  | Deconv (2,2,2) | (2,2,2) | - | 80-40 |
|  | Conv (3,3,3) | (1,1,1) | (1,1,1) | 320-320 | B11 | Conv (3,3,3) | (1,1,1) | (1,1,1) | 80-40 |
| B6 | Conv (3,3,3) | (2,2,2) | (1,1,1) | 320-320 |  | Conv (3,3,3) | (1,1,1) | (1,1,1) | 40-40 |
|  | Conv (3,3,3) | (1,1,1) | (1,1,1) | 320-320 |  | Deconv (2,2,1) | (2,2,1) | - | 40-20 |
| B7 | Deconv (2,2,2) | (2,2,2) | - | 320-320 | B12 | Conv (3,3,3) | (1,1,1) | (1,1,1) | 40-20 |
|  |  |  |  |  |  | Conv (3,3,3) | (1,1,1) | (1,1,1) | 20-20 |
|  |  |  |  |  |  | Conv (3,3,3) | (1,1,1) | (1,1,1) | 20-5 |
